# Impairment of blood-brain barrier is an early event in R6/2 mouse model of Huntington Disease

**DOI:** 10.1038/srep41316

**Published:** 2017-01-24

**Authors:** Alba Di Pardo, Enrico Amico, Francesco Scalabrì, Giuseppe Pepe, Salvatore Castaldo, Francesca Elifani, Luca Capocci, Claudia De Sanctis, Laura Comerci, Francesco Pompeo, Maurizio D’Esposito, Stefania Filosa, Stefania Crispi, Vittorio Maglione

**Affiliations:** 1 IRCCS Neuromed, Pozzilli, Italy; 2 Institute of Biosciences and Bioresources, IBBR, CNR, Napoli, Italy; 3 Institute of Genetics and Biophysics “A. Buzzati-Traverso”, Naples, Italy

## Abstract

Blood-brain barrier (BBB) breakdown, due to the concomitant disruption of the tight junctions (TJs), normally required for the maintenance of BBB function, and **to** the altered transport of molecules between blood and brain and vice-versa, has been suggested to significantly contribute to the development and progression of different brain disorders including Huntington’s disease (HD). Although the detrimental consequence the BBB breakdown may have in the clinical settings, the timing of its alteration remains elusive for many neurodegenerative diseases. In this study we demonstrate for the first time that BBB disruption in HD is not confined to established symptoms, but occurs early in the disease progression. Despite the obvious signs of impaired BBB permeability were only detectable in concomitance with the onset of the disease, signs of deranged TJs integrity occur precociously in the disease and precede the onset of overt symptoms. To our perspective this finding may add a new dimension to the horizons of pathological mechanisms underlying this devastating disease, however much remains to be elucidated for understanding how specific BBB drug targets can be approached in the future.

Huntington’s Disease (HD) is the most common inherited neurodegenerative disease characterized by uncontrolled excessive motor movements and cognitive and emotional deficits^1^. The disease-causing mutation is a CAG - trinucleotide repeat expansion exceeding the normal range in the *HTT* gene encoding the ubiquitous protein, huntingtin^2^.

Although the well-established underlying cause of HD, the disease presents an unprecedented complexity involving an as yet unspecified number of pathological factors that still remain to be fully elucidated. Besides the most commonly functional abnormalities described in HD pathology, it has been recently reported that the cerebral vascular system is also significantly disrupted in the disease^3^,^4^.

The disruption of the vasculature and the subsequent brain-blood barrier (BBB) breakdown has been strongly implicated in neurodegenerative disorders like Alzheimer (AD)^5^ and Parkinson disease (PD)^6^ however, it remains unknown whether such alterations represent initial events leading to neuronal cell death or whether it is a downstream consequence. The BBB is a highly dynamic and specialized structure that acts as biochemical barrier to protect the central nervous system (CNS) from systemic perturbations and to maintain the ideal condition for neurons to function properly^7^. Its key functional properties include the highly restrictive transbarrier transport, due to sealing of paracellular pathway by tight junctions (TJs), intricate complexes of transmembrane - occludins and claudins - and adaptor cytoplasmic - zona occludens-1 (ZO-1) - proteins^8^, and poor transcytotic traffic through caveolae^9^.

Increasing number of studies indicate that impaired BBB integrity may profoundly affect brain homeostasis and have far-reaching consequence on the overall health of the CNS^10^.

Recent studies have been conducted to investigate whether any potential dysfunctional and structural perturbation of BBB occurred during the development of the brain disorder also in HD^4^,^11^,^12^. In contrast to the first studies that failed to show any BBB leakage^11^,^12^, very recent data documented a major alteration of BBB permeability in severe disease symptomatic HD transgenic mice as well as in human patients^4^. The evidence of impaired BBB in HD may represent an important determinant of disease progression, however, the occurrence and the significance of the interplay between BBB disruption and HD pathology still remain to be completely elucidated.

Taking into account the obvious clinical and therapeutic relevance the BBB disruption may have in the complex pathophysiology of HD, we believe it is worth to further investigate this aspect and to understand how early the perturbation occurs in the disease. In the light of that and, based on the analogy existing between the pathophysiological profile of BBB perturbation in HD patients and symptomatic R6/2 mice^4^, we believe that a systematic study on BBB integrity in this mouse model, which recapitulates many features of human pathology at different stage of the disease^13^,^14^, may help to determine the potential early occurrence of BBB impairment, to delineate potential yet unexplored pathogenic processes and to develop more targeted therapeutic interventions.

Interestingly, although the timing of BBB breakdown remains still elusive for many of the neurodegenerative diseases, our findings, for the first time, clearly indicated that the very first signs of measurable BBB impairment in HD occur early in the disease, whereas alteration of gene expression levels of TJ proteins (TJPs) even precedes the onset of overt symptoms.

## Material and Methods

### Animal models

**Figure 1:**
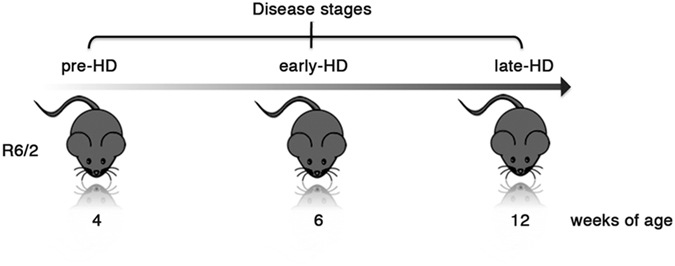
Schematic representation of age-related disease stage in transgenic HD R6/2 mice used in this study. Age-related disease stage referred to Carter *et al*.^14^.

All analyses were carried out in pre-symptomatic (pre-HD; 4 week old), early symptomatic (early-HD; 6 week old) and late symptomatic (late-HD; 12 week old) transgenic HD R6/2 mice (Fig. 1) and, in age and gender-matched wild-type (WT) control littermates. Briefly, a total of 54 mice (27 R6/2 and 27 WT) divided in two experimental groups were used in this study. The first set of mice (15 R6/2 - 5 mice per each disease stage and 12 age-matched WT mice - 4 mice per each corresponding age) was used for assessing BBB permeability by FITC-Albumin extravasation assay. The second set was indeed used for biochemical and gene expression analysis. R6/2 mouse colonies were maintained in the animal facility at IRCCS Neuromed. All animal studies were performed in accordance with approved protocols by the IRCCS Neuromed Animal Care Review Board and by “Istituto Superiore di Sanità” (permit number: 1163/2015- PR) and were conducted according to EU Directive 2010/63/EU for animal experiments.

### Evaluation of blood–brain barrier permeability

BBB integrity was evaluated using FITC-Albumin, a dye that is able to cross the barrier only when this latter is impaired^15^. Mice were infused with the dye (Sigma, 10 mg/ml in PBS at 10 ml/kg) into the jugular vein and twelve minutes after infusion, they were decapitated and the entire brain removed and frozen in cool isopentane (−80 °C). The brain was cut serially with a Jung CM1900 Cryostat (Leica Instruments, Germany) in 20 μm thick sections. The sections were observed with a 20× magnification under an Axiophot2 fluorescence microscope (Zeiss, Germany) equipped with a FITC filter. Twenty-four bit color pictures were acquired and analyzed using a digital camera system coupled to imaging software (Spot, Diagnostic Instruments, USA) under constant exposure time, gain and offset. Albumin extravasation was evaluated measuring selectively green fluorescence intensity corrected for background fluorescence and expressed as fluorescence arbitrary units (FAU). All images were taken in the first 30 s of light exposure, when no fluorescence decay was detected in preliminary studies. FITC-Albumin/laminin co-staining was performed by using a specific anti-laminin (pAb 1:1000 Novus Biological) antibody. Sections were then incubated with the respective secondary antibody conjugated to Cy3. Slides were then coverslipped with mounting medium (Vector) and scanned using a Nikon microscope.

### Brain tissue lysate preparation and immunoblotting

Mice at different disease stage were sacrificed by cervical dislocation and cortex and striatum from one half of the brain were dissected out, snap frozen in liquid N2 and pulverized in a mortar with a pestle. Tissues were homogenized in lysis buffer containing 20 mM Tris, pH 7.4, 1% Nonidet P-40, 1 mM EDTA, 20 mM NaF, 2 mM Na_3_V0_4_, and 1:1000 protease inhibitor mixture (Sigma-Aldrich) and sonicated with 2 × 10 s pulses. 20 μg of total protein lysate were resolved on SDS-PAGE and immunoblotted with anti-claudin-5 (1:1000) (Abcam). Anti-ß-actin (1:5000) (Sigma) was used for protein normalization. Protein bands were detected by ECL Plus and quantitated with Quantity One software (Bio-Rad Laboratories).

### RNA extraction and q-PCR

RNAs were isolated from the cortex and striatum from one half of the brain of R6/2 mice, at different disease stage, and of age-matched WT control mice using Trizol, (Life Technologies). Tissue homogenization was performed using Tissue lyser (Qiagen) following manufacturer instructions. RNA quality was assessed using Experion system (BioRad). For qPCR assays, from each sample 200 ng of total RNA were retro-transcribed using the High Capacity cDNA Reverse Transcription Kit (Applied Biosystem). q-PCR was performed by means of a 7900 HT Real Time PCR (Applied Biosystem). Gene specific primers for the selected genes (Table 1) were designed at exon-exon junctions using Primer express 2.0 (Applied Biosystems). mRNA expression levels of TJ proteins were normalized over Cyclophilin A, used as internal control. Normalized values in HD tissues were then expressed as fold of change of normalized values in WT tissues. The entire procedure for q-PCR analysis, including primer design, reactions, amplicon specificity, and determination of gene target expression levels, was performed as previously described^16^.

### Statistical analysis

Unpaired t-test or one-way ANOVA followed by Tukey post-hoc test, as appropriate, using a dedicated software (GraphPad Prism Software, version 5.0) was used to analyze data which were expressed as mean ± SD.

## Results

### Perturbation of BBB permeability occurs early in HD R6/2 mice

**Figure 2:**
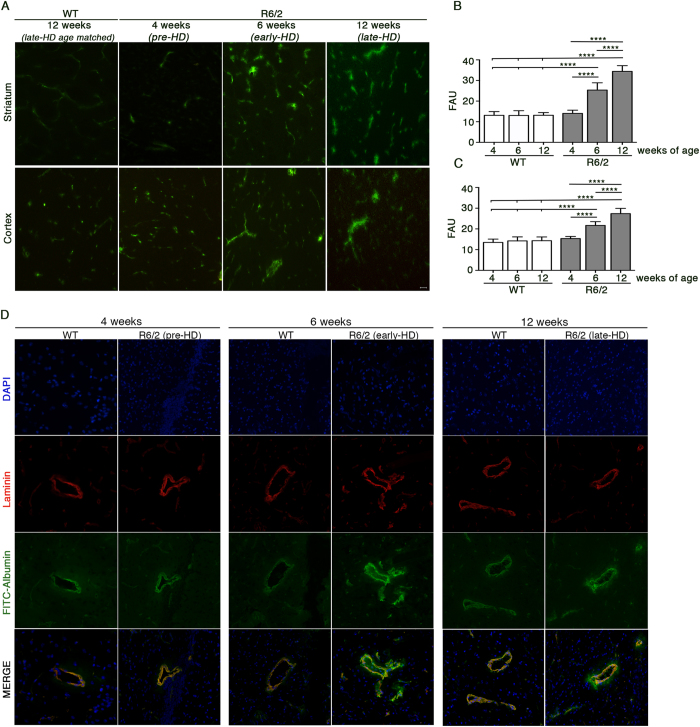
BBB disruption occurs early in HD pathogenesis in R6/2 mice. (**A**) Representative fluorescence micrographs of brain cryosections from striatum and cortex of R6/2 mice at different stage of the disease and from a representative late-HD age-matched WT littermate (12 week of age), showing FITC-Albumin extravasation (bright green fluorescence) in the brain parenchyma of early- and late-HD mice. Green fluorescence signals of FITC-Albumin in pre-symptomatic HD mice and WT control were only restricted to the inside of the brain blood vessels. (**B** and **C**) Graph shows quantitation of the green fluorescence emitted by FITC-Albumin, reported as Fluoresecence Arbitrary Unit (FAU), in the striatum and cortex of WT and R6/2 mice at different disease stages. Data are represented as mean ± SD. ****p < 0.0001 (Two Way ANOVA, Tukey post-test). (**D**) Representative fluorescence micrographs of brain cryosections from striatum of R6/2 mice at different stage of the disease and age-matched WT littermates showing only partial co-localization of FITC-Albumin (bright green fluorescence) with vessel marker laminin (red) in tissues from early and late-HD stages. Scale bar in the merge monograph represents 100 μm.

Loss of BBB integrity has been previously described in advanced disease stage in HD^4^ however, its impairment throughout the development of the disease still remains to be fully elucidated. To this regard, in order to evaluate how early in the disease the first signs of BBB leakage appear, tissue fluorescence for the permeability tracer FITC-Albumin was analyzed by fluorescence microscopy on freshly frozen brain tissue sections in pre-symptomatic condition as well as in early and late disease stages. Interestingly, when R6/2 brain tissues (striatum and cortex) were analyzed, a diffuse fluorescence was observed along perivascular spaces and brain parenchyma starting immediately after the very first signs of disease appeared (early-HD, 6 weeks of age) and persisted up to the advanced disease stage (late-HD, 12 weeks of age) (Fig. 2A–D). No appreciable sign of FITC-Albumin extravasation was visible in brain section from pre-symptomatic R6/2 mice (pre-HD, 4 weeks of age) (Fig. 2A) whose fluorescence pattern was similar to WT controls (Fig. 2A). Co-staining with the vascular marker, laminin, nicely confirmed the FITC-Albumin leakage in the parenchyma of HD brains (Fig. 2D) in both early and late stages of the disease (Fig. 2D). As expected, in WT control mice FITC-Albumin was restricted to the inside of the brain blood vessels and no signal in the surrounding brain parenchyma was detectable at any age (Fig. 2D).

### Alteration in the gene expression of TJ proteins in R6/2 mice occurs even before any disease signs appear

**Figure 3:**
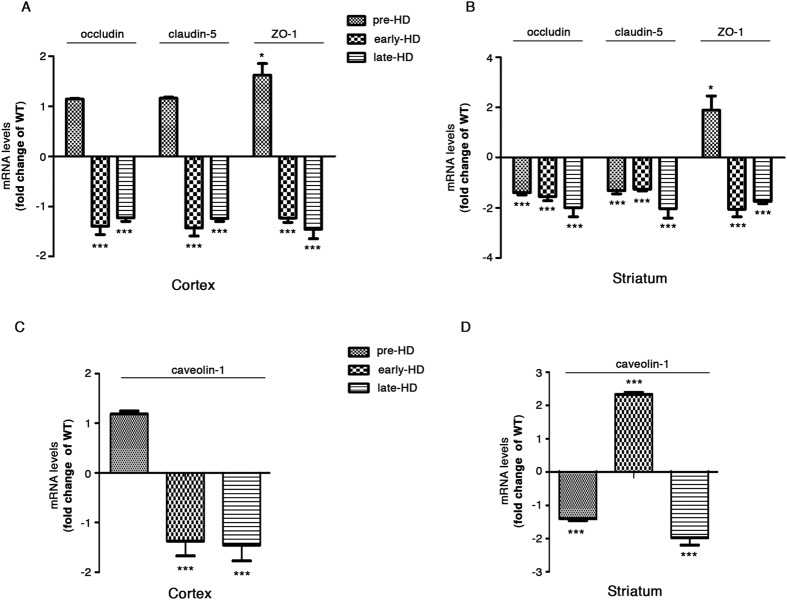
Alteration in the gene expression of TJ proteins in R6/2 mice occurs at pre-symptomatic stage of the disease. **(A** and **B)** Quantitative analysis of mRNA expression levels of TJPs - occludin, claudin-5 and ZO-1 and **(C** and **D)** of transcytosis-regulating protein caveolin-1 showing transcriptional deregulation early in the disease in both cortex and striatum isolated from pre-, early and late-HD R6/2 mice and age-matched WT littermates. Data are represented as mean ± SD, n = 4–5 for each group of mice. *p < 0.05; ***p < 0.0001, R6/2 mice versus age-matched WT littermates (Un-paired t-test).

In order to determine the molecular processes beyond BBB breakdown and the enhanced vascular permeability in HD, changes in the expression of genes encoding TJPs were evaluated in brain tissues of R6/2 mice at different disease stage**s**. Quantitative analysis of mRNA levels of the key endothelial TJ proteins, occludin and claudin-5 as well as of the TJ-associated adaptor protein, ZO-1, was performed in cortical and striatal tissues from pre-, early and late-HD R6/2 mice. Gene expression of all three TJ proteins was significantly down regulated in the cortical tissues of R6/2 mice starting from the early-HD stage of the disease (Fig. 3A). At the pre-symptomatic stage, while unchanged levels of occludin and claudin-5 mRNA were detectable in R6/2 mice with respect to age-matched WT littermates (Fig. 3A), transcript levels of ZO-1 were approximately 2-fold increased (Fig. 3A). Similar results were also found in the striatum of the same mice, however signs of deregulated occludin and claudin-5 mRNA levels were first detectable even before symptoms appeared (Fig. 3B).

In addition, the expression of caveolin-1, a scaffold protein implicated in the spatial organization of TJPs^17^ was also perturbed during BBB breakdown in HD (Fig. 3C,D). While in the cortical tissues caveolin-1 showed mRNA changes similar to that observed in the TJPs gene expression experiment (Fig. 3C), in the striatal counterpart a significant up-regulation was detected at early-symptomatic stage (early-HD) concomitantly to first traces of FITC-Albumin extravasation (Figs 2 and 3D).

### Protein expression of claudin-5 is significantly decreased in early symptomatic R6/2 mice

**Figure 4:**
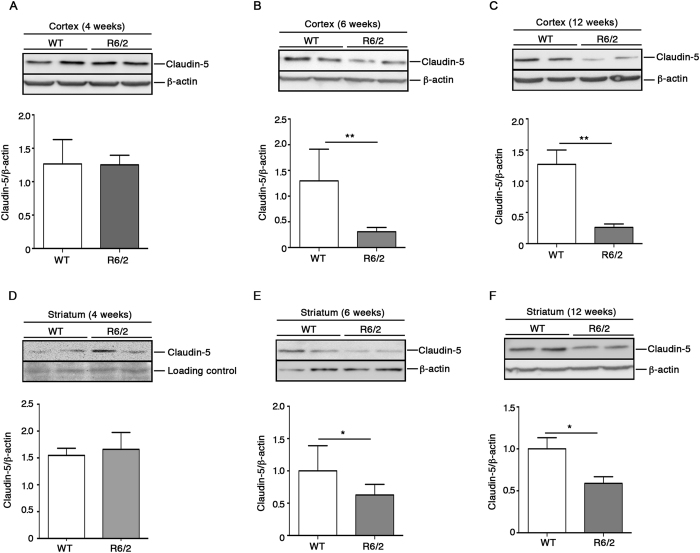
Expression of claudin-5 protein is reduced in brain tissues from early- and late-symptomatic R6/2 mice. Representative cropped immunoblottings and densitometric analysis of claudin-5 in cortical (**A**–**C**) and striatal (**D**–**F**) tissues isolated from pre- (**A** and **D**), early- (**B** and **E**) and late-HD (**C** and **F**) mice and age-matched WT littermates. Protein bands were visualized by ECL. Data are represented as mean ± SD, n = 4–5 for each group of mice. *p < 0.05; **p < 0.01 (Un-paired t-test).

Decrease expression of TJ proteins has been previously associated with increased permeability of BBB^18^. Claudin-5, which has been identified as the major integral membrane protein and primary seal of the TJs^19^,^20^, represents an early marker of endothelial dysfunction in different pathological conditions^21^,^22^. Here, with the aim of assessing whether the perturbed gene expression of TJPs (Fig. 3) observed in R6/2 mice at early disease stage, was associated with functional impairments of BBB, evaluation of claudin-5 protein expression was determined in both cortical and striatal tissues by western blotting. As shown in Fig. 4, analysis of claudin-5 in the cortex highlighted a protein expression profile that was coherent with the gene expression pattern observed at different stages of the disease. In particular, reduction of protein expression in R6/2 mice was first detectable early in the disease and persisted up to the late stages (Fig. 4B,C). Similar results were also observed in the striatal tissues of the same mice (Fig. 4D–F). No difference**s** were found between pre-HD R6/2 and age-matched WT control mice in any of the tissues analyzed (Fig. 4A).

## Discussion

Disruption of TJ and deregulated TJPs expression by disease or drugs can profoundly affect BBB^5^, whose disturbances are becoming a common denominator in different type of neurodegenerative disorders^23^,^24^ and whose dysfunctional permeability can lead to the leakage of various neurotoxic substances into the brain, resulting in neuronal damage and overall brain dysfunction^25^.

In the past years, several lines of evidence from studies in both animal models and humans have implicated abnormality of the BBB in the pathogenesis of many severe and progressive common neurological disorders^6^,^26^,^27^,^28^,^29^,^30^,^31^ and proposed BBB breakdown as an early pathological event in some of these conditions^32^,^33^. Because of the obvious detrimental repercussion the BBB breakdown may have on the overall CNS homeostasis and eventually on disease progression, it is urgent to test the hypothesis whether its impairment is an initial event also in the development of an un-curable rare neurological disease like HD. The recognition of BBB structural abnormalities is only recently becoming evident in HD^11^,^12^. However, aside from only one recent study reporting real evidence of BBB alterations in both symptomatic transgenic R6/2 mice and human patients^4^, a conclusive concept of BBB derangement throughout the disease course in HD is currently lacking. Thus, in this study, we sought to systematically explore the potential loss of BBB integrity at different stages as the disease progressed. To this purpose, given the similar pattern of BBB alteration in symptomatic R6/2 mice and moderate HD patients^4^, we further used this mouse line to test the hypothesis that BBB breakdown is an early event in HD. The choice of using R6/2 model was also supported by the evidence that despite the characteristic short and aggressive course of the disease, R6/2 mice exhibit a progressive HD-like behavioral and neuropathological phenotype that correspond to human HD^34^.

Interestingly, our results revealed for the first time that signs of TJs derangement and perturbed BBB homeostasis were first perceptible at very early stage of the disease even in absence of any overt symptoms as assessed by gene expression experiments in both cortex and striatum of R6/2 mice. In agreement with the evidence that BBB dysfunction is commonly associated with decreased TJPs expression^4^,^35^,^36^,^37^,^38^,^39^, in this study we demonstrated that such an association can be found early in the disease in this mouse model. Coherently, extravasation of FITC-Albumin in the brain parenchyma of early symptomatic R6/2 mice was paralleled by significant deregulation of TJPs expression at both mRNA and protein levels. mRNA and protein levels of claudin-5, the major functional constituent of TJs and a critical determinant of BBB integrity and paracellular permeability^40^, were found being significantly reduced starting from the early stage of the disease in the cortex of R6/2 mice. Similar gene expression profile was also detected for both occludin, mainly implicated in the formation of TJs as additional structure^40^ and for the TJs-associated protein, ZO-1. Conversely, in the striatum, signs of deregulated transcription of these genes were first detected at the pre-symptomatic stage before any obvious disease symptoms. While claudin-5 and occludin showed a comparable gene expression pattern, ZO-1 displayed a distinct behaviour at this stage. Based on the dynamic role ZO-1 has in the BBB stabilization and TJ assembly^40^,^41^,^42^ higher levels of mRNA in the pre-symptomatic R6/2 mice may represent an attempt of endothelial cells to counteract the disruption of TJs and the subsequent increase of paracellular permeability. In our perspective, although the discrepancy between cortical and striatal tissues, this finding may have a relevant pathophysiological significance and potentially define an important contributor of the selective and higher vulnerability of the striatum.

Moreover, in the light of previous evidence indicating that compromised BBB may be part of cascade of early pathologic events that eventually lead to cognitive decline and dementia in neurodegenerative disorders like AD^27^, here we speculate that a similar scenario may occur also in HD. In particular, perturbation of TJ protein gene expression, occurring before any detectable sign of BBB leakage in our model, may increase the vulnerability of the BBB and potentially represent a molecular event associated with the cognitive impairment that may precede motor symptoms in both pre-symptomatic R6/2 mice^43^ and prodromal HD subjects^44^,^45^.

Moreover, the finding of downregulated TJ proteins in the striatum of early symptomatic R6/2 mice along with a peculiar upregulation of caveolin-1, whose increase has been previously reported to precede the deregulation of structural TJPs in the early process of BBB breakdown^22^, are likely to reflect also increased transcytotic permeability in HD.

We claim that the novelty of our study lies in the finding of early functional impairment of BBB in HD and we believe that our data together with previous evidence^4^, may add a new dimension to the horizons of pathological mechanisms operating in this devastating disease.

Although our data need to be complemented by future studies focusing on the underlying molecular mechanisms and the understanding of the existing relationship between BBB perturbation and progress of neurodegeneration in HD, the present findings might contribute to figure out how specific BBB drug targets can be approached in the future. Collectively, to our perspective these findings in HD may represent an important aspect more intriguing than is generally believed. It may suggest important implications for disease pathogenesis and may help in formulating various new therapeutic strategies to protect barrier function for potentially extend functionality and lifespan also in HD patients.

## Additional Information

**How to cite this article**: Di Pardo, A. *et al*. Impairment of blood-brain barrier is an early event in R6/2 mouse model of Huntington Disease. *Sci. Rep.* **7**, 41316; doi: 10.1038/srep41316 (2017).

**Publisher's note:** Springer Nature remains neutral with regard to jurisdictional claims in published maps and institutional affiliations.

## Figures and Tables

**Table 1:** Genes amplified by q-PCR and sequences of the primers used for their amplification.

Gene		Primer sequence
Occludin	Forward	AGACCTGATGAATTCAAACCCAAT
	Reverse	ATGCATCTCTCCGCCATACAT
Caveolin 1	Forward	CGTAGACTCCGAGGGACATCTC
	Reverse	GGCTTGTAGATGTTGCCCTGTT
Claudin	Forward	CAGTTAAGGCACGGGTAGCA
	Reverse	GGCACCGTCGGATCATAGAA
ZO-1	Forward	TTCTTCGAGAAGCTGGATTCCT
	Reverse	TCTGGCAACATCAGCTATTGGT
Cyclophillin A	Forward	TCCAAAGACAGCAGAAAACTTTCG
	Reverse	TCTTCTTGCTGGTCTTGCCATTCC
